# Bilateral anterior and intermediate uveitis in SARS-CoV-2 associated multisystem inflammatory syndrome in a pediatric patient

**DOI:** 10.1186/s12969-022-00712-8

**Published:** 2022-07-30

**Authors:** Jessica Shantha, Amit K. Reddy, Amol Sura, Adrian Tsang, Kareem Moussa, Nisha Acharya, John Gonzales, Thuy Doan

**Affiliations:** 1 Francis I. Proctor Foundation, Department of Ophthalmology, University of California San Francisco, 490 Illinois Street, CA 94158 San Francisco, USA; 2 Department of Ophthalmology, University of Colorado School of Medicine, 1675 North Aurora Court F731, CO 80045 Aurora, USA; 3 Department of Ophthalmology, Duke University, 2351 Erwin Rd, NC 27705- 4699 Durham, USA; 4 Davis Department of Ophthalmology & Vision Science, University of California, 4860 Y Street, Suite 2400, 490 Illinois Street, Floor 2, CA 95817, 94158 Sacramento, San Francisco, USA

**Keywords:** COVID-19, Uveitis, Multisystem inflammatory syndrome in children

## Abstract

**Purpose:**

To report a case of bilateral anterior intermediate uveitis after recovery from SARS-CoV-2 associated multisystem inflammatory syndrome in children (MIS-C).

**Case Report:**

A 9-year-old male presented with bilateral anterior intermediate uveitis with fluorescein angiography (FA) leakage of the disc and peripheral vasculature 1 month after recovery from MIS-C. He was treated with difluprednate 0.05% in both eyes with resolution of FA leakage, but our patient has required an extended treatment of topical therapy and the need long term immunosuppression.

**Conclusions:**

This is a case of uveitis presenting after recent MIS-C related to SARS-CoV-2. Ongoing follow up and monitoring is required, and it is important for the ophthalmologist and rheumatologist to be aware of this rare complication during the current COVID-19 pandemic.

## Background

Multisystem inflammatory syndrome in children (MIS-C) has emerged as a rare clinical entity in pediatric patients with recent exposure to severe acute respiratory syndrome coronavirus 2 (SARS-CoV-2). Patients can present with a myriad of findings including fever, mucocutaneous changes, cardiac conduction abnormalities, myocardial dysfunction, gastrointestinal abnormalities, and neurological changes with nonspecific laboratory derangements in inflammatory and coagulation pathways. MIS-C is thought to be distinctly different from Kawasaki disease (KD) although overlap in clinical presentation does exist. The American College of Rheumatology diagnostic criteria and consensus guidelines for the diagnosis and management of MIS-C associated with SARS-CoV-2 includes conjunctivitis as a clinical feature [1]. Ophthalmic manifestations associated with SARS-CoV-2 are increasingly recognized in adults and ranges from mild conjunctivitis and retinopathy to retinal artery and vein occlusions and uveitis, but these features are rarely noted in children [2]. Herein we report a case of bilateral anterior intermediate uveitis in a pediatric patient with recent MIS-C.

## Case Report

**Fig. 1:**
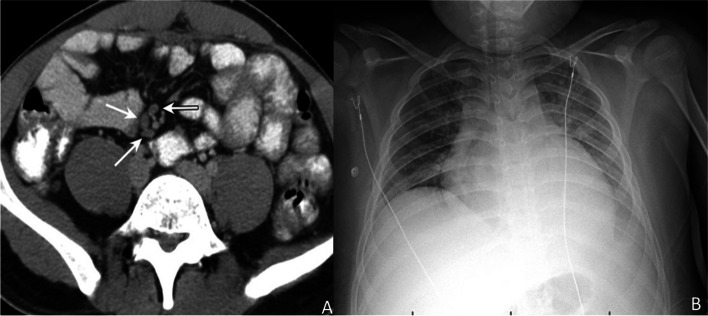
Computerized tomography scan of the abdomen (**A**) revealed lymphadenopathy and chest x-ray (**B**) showed bilateral pulmonary edema and pleural effusions

A 9-year-old male with a past medical history of obesity, asthma, and coronavirus (COVID-19) infection one-month prior requiring hospitalization presented with fever, chills, myalgias, right eye conjunctivitis, cracked edematous lips, abdominal pain, macular rash on back and knees, and lower extremity edema. Laboratory evaluation revealed anemia (Hemoglobin 8.0 gm/dl, normal:11.5-15.5 g/dl, Hematocrit 25%, normal: 35–45%), thrombocytopenia (103 Thousand/µL, normal:140–400 Thousand/µL), and elevated c-reactive protein (185.4 mg/L, Normal < 8 mg/L), erythrocyte sedimentation rate (20 mm/hr, normal: <15 mm/hr), fibrin d-dimer (1.44, normal:<0.25), functional fibrinogen (412 mg/dl, normal: 199–409 mg/dL), activated partial thromboplastin time (56.7 s, normal: 27.0-37.2 s), and prothrombin time (17.2 s, normal: 11.7–15.1 s). Imaging studies obtained at admission included a normal chest x-ray, a normal echocardiogram, and an abnormal CT of the abdomen that displayed right lower quadrant lymphadenopathy (Fig. 1). Antibody testing to the spike protein of SARS-CoV-2 was detected with no prior history of vaccination. He was diagnosed with MIS-C secondary to SARS-CoV-2. He was initially treated with intravenous immune globulin and low dose aspirin. Throughout his hospital stay he required a multidisciplinary team of specialists: rheumatology, hematology, cardiology, infectious disease, and nephrology. His condition worsened, and he developed anasarca, pulmonary edema, bilateral pleural effusion (Fig. 1), respiratory distress, acute kidney injury, and mild ectasia of 2 coronary arteries on repeat echocardiogram. Eventually, he required admission to the pediatric intensive care unit for high flow nasal canula oxygen, intravenous high dose methylprednisolone, and fluid diuresis with furosemide. He was admitted for a total of 11 days and recovered well with resolution of his anasarca, pulmonary edema, acute kidney injury, and ectasia of his coronary arteries. The patient was discharged on 30 mg prednisone daily and low dose aspirin with planned follow up with rheumatology and cardiology.

**Fig. 2:**
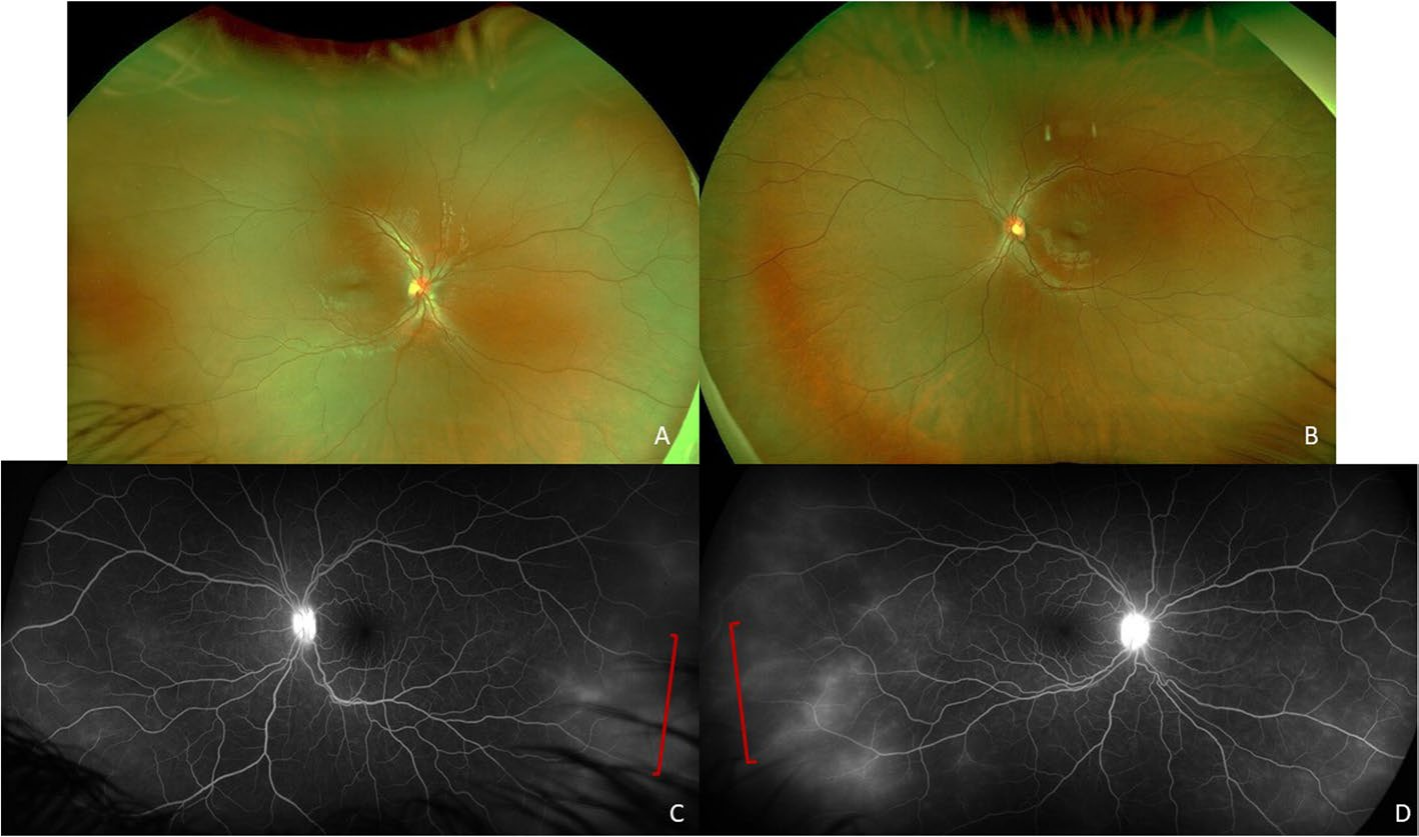
Posterior segment imaging of both eyes with normal fundus photos of the right (**A**) and left (**B**) eye. Fluorescein angiography of the right (**C**) and left eye (**D**) show disc leakage and late peripheral vascular leakage (brackets) in both eyes

One month after discharge, he noted left eye redness and photophobia after he self-discontinued prednisone, which prompted a referral to pediatric ophthalmology. On initial exam his vision was 20/20 in the right eye and 20/50 in the left eye with normal intraocular pressures in both eyes. Anterior segment exam was normal in the right eye and showed 2 + conjunctival injection with 2 + anterior chamber cell and posterior synechiae in the left eye. Exam of the posterior segment was limited, and the patient was diagnosed with unilateral non-granulomatous anterior uveitis and started on prednisolone acetate 1% every hour and cyclopentolate two times per day. A few days later he complained of photophobia in the right eye and had an exam consistent with anterior uveitis with 1 + conjunctival injection, 2 + anterior chamber cell and flare, and posterior synechiae. Treatment was initiated with prednisolone acetate 1% every hour and cyclopentolate two times per day in the right eye and continued in the left eye. A limited laboratory work up was initiated and referral was placed for further evaluation to the Proctor Foundation at the University of California San Francisco. Three weeks later he was examined at the Proctor Foundation and exam revealed a visual acuity of 20/20 and 20/25 in the right and left eye respectively and normal intraocular pressure in both eyes. Anterior segment exam showed white conjunctiva and 3 + anterior chamber cell, and 1 + cell in the anterior vitreous in both eyes. Fluorescein angiography (FA) was performed which revealed disc leakage and peripheral vascular leakage in both eyes (Fig. 2). A diagnosis of bilateral non-granulomatous anterior and intermediate uveitis was made. Prednisolone acetate 1% was discontinued and difluprednate 0.05% 4 times a day was initiated in both eyes with continuation of cycloplegic medication. Laboratory investigations were notable for positive antinuclear antibody (1:40, negative < 1:40); interferon gamma release assay (QuantiFERON Gold), Bartonella antibodies, Treponemal antibodies, urine Beta-2-microglobulin, and HLA-B27 were negative. Throughout our patient’s treatment course, he developed mild ocular hypertension in both eyes (intraocular pressure elevation to 24, normal < 21) and was started on dorzolamide 2% 2 times/day in both eyes with a good response. He was able to taper off the dufluprednate 0.05% and transition to prednisolone acetate 1% daily in both eyes with discontinuation of cycloplegic drops. At last follow up, 9 months after initial presentation, his vision was 20/20 in the right eye and 20/25 in the left eye with normal intraocular pressure. Right eye exam showed 2 + anterior chamber cell, with broken posterior synechiae, a small posterior subcapsular cataract, and rare anterior vitreous cell. The left eye exam revealed 1 + anterior chamber cell exam broken posterior synechiae, a small posterior subcapsular cataract and rare anterior vitreous cell. Posterior segment exam was normal and repeat FA showed resolution of disc and vascular leakage. His topical prednisolone acetate 1% was increased to 4 times/day and dorzolamide 2% 2 times/day was continued in both eyes. We initiated methotrexate 20 mg weekly with folic acid 1 mg daily given the multiple recurrences (3 total) of inflammation in both eyes with the development of complications of inflammation and topical treatment including ocular hypertension and posterior subcapsular cataracts.

## Discussion

This is a case of bilateral anterior and intermediate uveitis that was temporally associated with recent MIS-C.Very few ophthalmic complications have been reported in pediatric patients with active or recent COVID-19 infection which include conjunctivitis and uveitis [2–6]. Uveitis is becoming increasingly recognized as a complication in MIS-C with punctate epitheliopathy, scleritis, anterior uveitis, and disc edema reported in case reports and series. In these cases, children presented with eye symptoms at initial evaluation for MIS-C and within 7–10 days of their hospital admission. Treatment ranges from topical prednisolone acetate to intravenous and oral corticosteroids [2–6]. Our patient, however, presented a month after MIS-C recovery with ocular symptoms and bilateral anterior intermediate uveitis. In addition, ophthalmic imaging revealed angiographic leakage in both eyes which is a unique feature to this case presentation. Given that an ophthalmic exam was not performed at the time of his hospitalization, it is hard to know if indeed he initially presented with an anterior uveitis that was misdiagnosed as a conjunctivitis. His treatment has required long term topical corticosteroids with the development of ocular complications and persistent bilateral inflammation which has prompted initiation of systemic immunosuppression with methotrexate. In contrast to Juvenile Idiopathic Arthritis associated anterior uveitis, which is usually asymptomatic our patient was very symptomatic making this distinctively different. As children may have idiopathic uveitis without a known eliciting infection or other systemic disease, this also could be a possibility in our patient and while temporally associated with MIS-C we cannot say with complete certainty it was the cause.

In adult patients, uveitis has been diagnosed during acute SARS-CoV-2 infection and during disease convalescence. Particularly, in one case a diagnosis of bilateral intermediate and posterior uveitis was made 6 weeks after acute SARS-CoV-2 infection. Oral prednisone therapy was initiated and adequately treated this disease, but this patient flared and was treated with long term immunosuppression with azathioprine [7].

Uveitis is a known manifestation of Kawasaki disease and can be present in 29–83% of patients [8]. Given the similarities in clinical presentation and potential overlapping mechanisms in pathogenesis that includes a hyperinflammatory syndrome, it is not surprising that uveitis is being increasingly recognized in MIS-C. Uveitis can develop over time and may be implicated in COVID-19 ‘long haulers’. It is important to be vigilant of this complication and ocular manifestations temporally associated with SARS-CoV-2 infection.

## Data Availability

Not applicable.

## Abbreviations

MIS-C: Multisystem inflammatory syndrome in children
KD: Kawasaki disease
SARS-CoV-2: Severe acute respiratory syndrome coronavirus 2
COVID-19: Coronavirus disease 2019
